# Enhancing growth and salinity stress tolerance of date palm using *Piriformospora indica*

**DOI:** 10.3389/fpls.2022.1037273

**Published:** 2022-11-25

**Authors:** Miloofer Sabeem, Mughair Abdul Aziz, Sangeeta K. Mullath, Faical Brini, Hatem Rouached, Khaled Masmoudi

**Affiliations:** ^1^ Department of Integrative Agriculture, College of Agriculture and Veterinary Medicine, United Arab Emirates University, Al−Ain, Abu−Dhabi, United Arab Emirates; ^2^ Department of Vegetable Science, College of Agriculture, Kerala Agricultural University, Vellanikkara, Thrissur, India; ^3^ Plant Protection Laboratory, Center of Biotechnology, Sfax (CBS), University of Sfax, Sfax, Tunisia; ^4^ Michigan State University, Plant and Soil Science Building, East Lansing, MI, United States

**Keywords:** Antioxidant, date palm, molecular mechanism, Na^+^/K^+^ ratio, *P. indica*, salt stress

## Abstract

Endophytic fungi are known to enhance plant growth and performance under salt stress. The current study investigated the growth, as well as biochemical and molecular properties of *Phoenix dactylifera* colonized with the mutualistic fungus *Piriformospora indica*, under control and salinity stress. Our findings indicated an increase in the plant biomass, lateral root density, and chlorophyll content of *P. indica*-colonized plants under both normal and salt stress conditions. Furthermore, there was a decline in the inoculated plants leaf and root Na^+^/K^+^ ratio. The colonization enhanced the levels of antioxidant enzymes such as catalase, superoxide dismutase, and peroxidase in plants. Increased ionic content of Zn and P were also found in salt-stressed date palm. The fungus colonization was also associated with altered expression levels of essential Na^+^ and K^+^ ion channels in roots like *HKT1;5* and *SOS1* genes. This alteration improved plant growth due to their preservation of Na^+^ and K^+^ ions balanced homeostasis under salinity stress. Moreover, it was confirmed that *RSA1* and *LEA2* genes were highly expressed in salt-stressed and colonized plant roots and leaves, respectively. The current study exploited *P. indica* as an effective natural salt stress modulator to ameliorate salinity tolerance in plants.

## Introduction

*Piriformospora indica* (*P. indica*) is a basidiomycete of the Sebacinales order. It is widely found as a harmless root endophyte and is extremely versatile due to its mycorrhizal association to promote plant growth (Kundu and Vadassery, 2022). It impacts growth and development through its imitation of root Arbuscular Mycorrhizal Fungi (AMF) functions such as bioregulation, biofertilization (nutrient exchange), and bioprotection (tolerance and resistance to abiotic and biotic stresses). The property that *P. indica* has a broad range of hosts led to the assumption regarding its potential application as a symbiotic microbe essential for enhancing plant yield and productivity in sustainable agriculture. The mechanisms behind its beneficial effects have been interpreted using different model interaction systems such as *P. indica*–*Arabidopsis thaliana* and *P. indica*–*Hordeum vulgare* (Achatz et al., 2010; Qiang et al., 2012). In contrast to AMF, *P. indica* can be easily cultivated in axenic culture, where it asexually produces a large number of thick-walled, pear-shaped spores called chlamydospores, which have a longer shelf-life.

*P. indica* can efficiently coexist with plants threatened by salinity stress. The concentration of harmful Na^+^ ions in plants increases due to excess of salt in the root zone, which limits the water uptake. This increases damage to the membrane integrity, causes reactive oxygen species (ROS) build-up, and results in imbalanced nutrient uptake. Consequently, it negatively impacts the photosynthetic capacity and alters cellular metabolism. This reduces growth and crop yield, as well as causes senescence (Li et al., 2022b). Plants use a variety of composite sensing, signaling, and response mechanism to regulate their tolerance for alleviating the negative damages of salt stress (Hanin et al., 2016).

These mechanisms include the ion compartmentalization in vacuoles, release of compatible solutes, and counterbalancing of ROS. Another pathway that plants have developed to cope with increased levels of tissue and cellular Na^+^ ions is homeostasis through Na^+^ transport regulation. To achieve ion homeostasis, sodium extrusion at the root–soil interface, as well as some level of Na^+^ efflux in every other cell type, is presumed to be of critical importance for improving the salt tolerance of glycophytes (Pardo et al., 2006). Sodium extrusion at the root surface is not the only mechanism through which plants counter sodium accumulation in the cell cytosol. In fact, sodium sequestration into the vacuole is aided by the tonoplast Na^+^/H^+^ antiporter (NHX)driven by the vacuolar H^+^-ATPase and H^+^-pyrophosphatase. These are important regulators of intracellular ion homeostasis that attenuate the toxic levels of cytoplasmic Na^+^, enhance K^+^ uptake, and are critical for cell expansion and plant stress acclimation (Bassil et al., 2019). High-affinity K^+^ transporter (HKT) is involved in encoding for Na^+^ and/or K^+^ transporters at the cell plasma membrane. These transporters control the movement of Na^+^ ions across tissues and are essential for improving salt tolerance in several plant species (Ali et al., 2021).

Symbiotic associations between plants and beneficial fungus such as *P. indica* alters plant secondary metabolism, promotes plant growth, and reduces the damages of abiotic stresses and enhances plants fitness by modulating their stress tolerance mechanisms (Li et al., 2022a). However, cell wall act as a barrier for beneficial microorganism’s entry into plant tissues, which is overcome through the secretion of cell wall-destroying enzymes (CWDE). The activity of *P. indica* CWDE destroys plant cell wall polysaccharides for an entry pathway to plant cells (Boorboori and Zhang, 2022). In extremely arid conditions, date palm, *Phoenix dactylifera*remains viable in saline soils and soils with low water supply (Müller et al., 2017; Hazzouri et al., 2020). They can grow in soils irrigated with 12 dS m^-1^ salinity level, exhibiting low to no symptoms of salt stress (Ramoliya and Pandey, 2003). However, date palms have been reported to encounter a 3.6% reduction in their yield with every unit increase in salinity above 4 dS m^-1^ (Maas and Grattan, 1999). This has caused a reduction in productivity, crop failures, and abandonment of farms in severe salt-affected areas. Thus, *P. indica* can play a mutualistic role with date palm to improve the latter’s productivity in high salt-stressed soils.

The impact of salinity stress on date palm is similar to other plants. Al Kharusi et al. (2017) demonstrated a high concentration of Na^+^ ions in roots and shoots, reduced K^+^ ions in the shoot, declined leaf relative water content, and increased electrolyte leakage in sensitive varieties. The function of date palm salinity responsive genes was studied by Patankar et al. (2019), who showed that the expression of the *P. dactylifera* metallothionein gene (*PdMT2A*) in *Saccharomyces cerevisiae*, a salinity sensitive yeast mutant, enhanced its drought, oxidative, and salinity stresses tolerance. Moreover, the induced expression of *PdMT2A* in transgenic Arabidopsis plants reduced the accumulation of toxic Na^+^ ions and maintained higher K^+^/Na^+^ ratio in comparison to wild type. These changes were attributed to the regulatory role of the transgene on HKT transporters. *P. indica* triggers various salt-responsive genes associated with stress adaptation and cellular growth improvement during salt stress. Discovering genes and pathways involved in metabolic and molecular processes, such as ion homeostasis, antioxidant activities, and cell wall integrity are of particular importance to unravel the mechanism by which *P. indica* can trigger salt tolerance in date palm and to elucidate the molecular mechanisms related to stress adaptation and functional genomics studies.

The *P. Indica’s* influence on date palm salt stress tolerance mechanism have not been examined so far. Thus, the present study was aimed to investigate the potential role of *P. indica* in promoting the growth and ameliorating the salinity tolerance of date palm. This study was designed to assess the growth-related parameters of date palm seedlings colonized or not with *P. indica* under normal or salt stress conditions. The Na^+^/K^+^ ratio, which is an index to discriminate between sodium and potassium, was exploited as a selection tool to screen for salinity tolerance. Furthermore, antioxidant activity was explored as the tolerance mechanisms that *P. indica* triggers under salinity stress. In addition, the current study analysed the transcription level of several salt stress responsive genes in roots and leaves of inoculated and non-inoculated date palm seedlings.

## Material and methods

### Plant material and growth conditions

Tissue culture seedlings of the Khalas variety maintained in ½ MS liquid medium were obtained from the date palm tissue culture laboratory of UAE University. The plants were maintained in a growth chamber at 25°C under a16 h light/8 h dark photoperiod at 60% relative humidity for four weeks before any treatment. For growing plants in soil, established seedlings with good rooting systems were transplanted into torpedo pots (5 cm in diameter and 18 cm in length) containing peat moss and grown in a growth chamber in similar conditions. The plants were not provided with any fertilizers in the pots.

### Fungal growth and root colonization

*P. indica* was cultivated on potato dextrose agar (PDA) media (Sigma, St. Louis, USA) and incubated at 28°C in the dark for 10 days. For liquid culture, chlamydospores were collected from the agar plate and used to inoculate 100 ml of Kaefer medium (Kaefer, 1977) in 500 ml Erlenmeyer flasks, which were then incubated at 28°C on a rotary shaker at a speed of 100 rpm for 8–10 days. The *P. indica* liquid culture was harvested by centrifugation, and mycelium was washed three times with distilled-sterilized water. Co-cultivation of *P. indica* with roots of Khalas date palm seedlings grown *in vitro* was carried out by injecting 500 µl of diluted mycelial solution (1%) into the media near the roots and gently shaking the mixture. The inoculated seedlings were maintained in a growth chamber at 25°C with a 16 h/8 h light–dark cycle. The bottom of the tubes containing the seedlings were covered with aluminium foil to limit the light reaching the root zone of the seedlings. On the other hand, the control plants received only water and were maintained under the same conditions. For inoculating *P. indica* into the soil, 2 ml of 1% suspended mycelium was added into the root rhizosphere one and two weeks after transplanting. The soils were also mock treated with water. The suspension of mycelium that was used for both *in vitro* and in soil experiments had 1.5 × 10^6^ spores per ml.

### Salt treatment

After three weeks of co-propagation of *P. indica* with roots of Khalas date palm seedlings grown *in vitro*, the seedlings were transferred to ½ MS medium having 250 mM NaCl, while no salt was added to the normal condition grown plants. The seedlings were grown under the same conditions for 20 additional days and harvested for downstream analysis. After three weeks of inoculation with *P. indica*, the soil experiment was performed by placing pots of colonized and non-colonized plants separately in trays immersed in water containing 250 mM NaCl for 20 days and harvested for downstream analysis. Following this, different parameters determining salt stress tolerance, such as plant growth, biomass, ion homeostasis, and antioxidant enzymes activities, were determined.

### Root architecture and observation of *P. indica* colonization

Predominant root length and lateral root density were examined in the seedlings propagated on soil pots with and without *P. indica* for 20 days under control and salinity stress conditions. The pictures of the date palm seedlings were analysed using ImageJ, a free image processing and analysis program (https://www.imagej.nih.gov/ij/download.html). Root segments of the colonized plants were cut uniformly into 1-cm-long pieces. The root samples were boiled in 10% KOH for 5 min, washed with sterile water, and then neutralized with 2% HCl. The roots were then stained with 0.5% trypan blue in lactophenol for 10 min. Afterward, they were destained using lactophenol solution for 15 min to remove excess stain. The stained root bits were viewed under a compound bright field microscope and assessed for colonization density.

### Measurement of Na^+^ and K^+^ ion concentration

Leaf and root samples were harvested from seedlings grown in soil with and without *P. indica* under normal and salt stress conditions for 20 days and shrivelled at 80°C for a day in oven. The extraction of Na^+^ and K^+^ ions were performed using complete digestion of the dried tissue on 1% HNO_3_ overnight at 60°C. The Inductively Coupled Plasma Emission Optical Spectrophotometer (ICP-OES, Perkin Elmer) was used to measure the Na^+^ and K^+^ ions concentrations.

### Determination of total chlorophyll content

To measure the chlorophyll concentration of the different samples, fresh leaves were used from the date palm seedlings after 20 days. Three leaves from three different plants for each treatment were harvested to measure chlorophyll content. 0.5 g of fresh plant leaf was placed in a mortar, mixed and squashed with 80% acetone. The chlorophyll was efficiently extracted with repeated grinding for several times. The centrifugation of the extract was performed at 2500 g for 5 min. The obtained supernatant absorbance was evaluated at 646 and 663 nm. The following formula in mg g^-1^ (FM), was used to measure each sample total chlorophyll content: [(7.15 x OD663) + (18.71 x OD646)] V/M, where V indicates the total extracts volume per litre and M corresponds to the fresh material mass (FM).

### Antioxidant enzymatic assays

Protein extraction and enzymatic assays were conducted with the optimized protocols reported by Benhiba et al. (2015). Leaf and root fresh material (500 mg) of control and stressed plants were ground to a fine powder with liquid nitrogen and then homogenized with 5 ml of extraction buffer containing 0.1 M potassium phosphate buffer (pH 7.0), 0.1g polyvinylpolypyrrolidone (PVPP), 0.1 mM EDTA, and 0.5 mM PMSF (phenyl methyl sulfonate fluoride). The homogenate was centrifuged at 18,000 x g for 10 min at 4°C. The supernatant was used for assaying the enzyme activity. The protein concentration in the different extracts was measured using bovine serum albumin (BSA) as a standard with the protocol described by Bradford (1976). The activity of Catalase (CAT, EC 1.11.1.6) was measured by following the consumption and disappearance of H_2_O_2_ at OD = 240 nm (Aebi, 1984). The reaction solution contained 0.1 M potassium phosphate buffer (pH 7.0), 20 mM H_2_O_2_, 0.1 mM EDTA, and 100 µl of extracted enzyme in a 2 mL volume. Superoxide dismutase (SOD, EC 1.15.1.1) activity was estimated by measuring its ability to impede the photochemical reduction of nitro blue tetrazolium (NBT) and the method of Beyer and Fridovich (1987) was used for the assay. The amount of enzyme leading to a 50% inhibition of NBT reduction at 25°C was defined as one unit of SOD. The activity of SOD was indicated as unit min^-1^ mg^-1^ protein. The activity of Peroxidase (POD, EC 1.11.1.7) was determined with the guaiacol test by using the change of absorbance, OD = 470 nm. The assay of the activity was performed for 3 min in a reaction mixture consisting of 100 mM potassium phosphate buffer at pH 7.0, 10 mM H_2_O_2_, 20 mM guaiacol, and 0.1 mL of enzyme extract in a 3 mL volume (Polle et al., 1994).

### RNA extraction and quantitative real-time PCR analysis

Total RNA from roots and leaves of control and salt-stressed plants with 250 mM NaCl for 20 days, was extracted from the *P. indica* inoculated and non-inoculated seedlings using the RNeasy total RNA isolation kit (Qiagen). For this, 1 µg of total RNA was treated with RNase-free DNase I and reverse-transcribed to cDNA using the SuperScript III Reverse transcriptase kit (Invitrogen) in accordance with the manufacturer’s instructions. Quantitative real-time PCR was performed using SYBRTM Select Master Mix (Applied Biosystems) in 96-well plates with Applied Biosystems machine of Real-Time PCR. The reactions for the PCR were performed in a 10 μl terminal volume consisting of 3 μl cDNA (acquired from 40 ng of DNase-treated RNA), primers of 0.5 μl (at 10 μM), master mix of 5 μl 2x SYBR Green I master mix, and RNase-free water (Sigma) of 1 μl. The reaction involved an initial denaturation for 10 min at 94°C followed by 45 cycles with 10 s at 94°C, 10 s at 60°C, and 15 s at 72°C, then a liquification curve with 5 s at 95°C, 1 min at 65°C, and 5 min, with temperature rising from 65°C to 97°C. Primers used for real-time PCR were designed by the Primer 3 Input software and are presented in **Table 1**. The relative expression level was measured using the formula: 2^-ΔΔCT^, where ΔΔCT corresponds to (CT of Target gene – CT of Actin gene) under stressed condition – (CT of Target gene – CT of Actin gene) under control condition.

**Table 1:** Sequences of oligonucleotides used in real-time PCR expression analysis.

Primers	Nucleotide sequences (5’–3’)	Accession no.
Date palm *Actin* gene		XM_008778129.4
*Actin* F	GCGATTCAGGCAGTTCTTTC	
*Actin* R	AATTTCCCGTTCTGCAGTTG	
Date palm *HKT1;5* gene		XM_008783694.4
*HKT1;5* F	CAAAATGCTGTAGCGAGCAA	
*HKT1;5* R	TTTTCTCTTGCTGCCACCTT	
Date palm *SOS1* gene		XM_026807192.2
*SOS1* F	TGCTTAGCTGGCCTGAAAAT	
*SOS1* R	GTATGACAAGCTGCGCGTAA	
Date palm *RSA1* gene		XM_008782850.3
*RSA1* F	CAAAATGCTGTAGCGAGCAA	
*RSA1* R	TCACCTGACCTGCTCTCCTT	
Date palm *LEA2* gene		XM_039120165.1
*LEA2* F	TCGGCATCCTCTACCTCATC	
*LEA2* R	CGATTTTCTTGTTCGGGTTC	

### Statistical analysis

Statistical parameters such as mean and standard deviation (SD) were measured for the data responses of date palm seedlings under control and stress condition. Each of the experiments were conducted with three biological replicates, using three technical repetitions. Data were analysed using Student’s independent t-test for significance in terms of the difference between *P. indica*-inoculated and non-inoculated plants under control and salt stress conditions. The Shapiro-Wilk test was performed for analysing distribution of data normality, and the homoscedasticity of data was evaluated using Levene’s test. The analyses were performed using the R statistical software.

## Results

### *P. indica* stimulates the growth of date palm seedling under normal and salt stress conditions

The presence of mutualistic fungus, *P. indica* in date palm roots of the Khalas variety grown *in vitro* was confirmed by observation of stained root bits under a compound bright field microscope and PCR amplification of Pitef1 (translation elongation factor EF-1α) gene at 20 days after inoculation (primers Pitef Fw: TCGTCGCTGTCAACAAGATG; Pitef Rv: GAGGGCTCGAGCATGTTGT).

**Figure 1:**
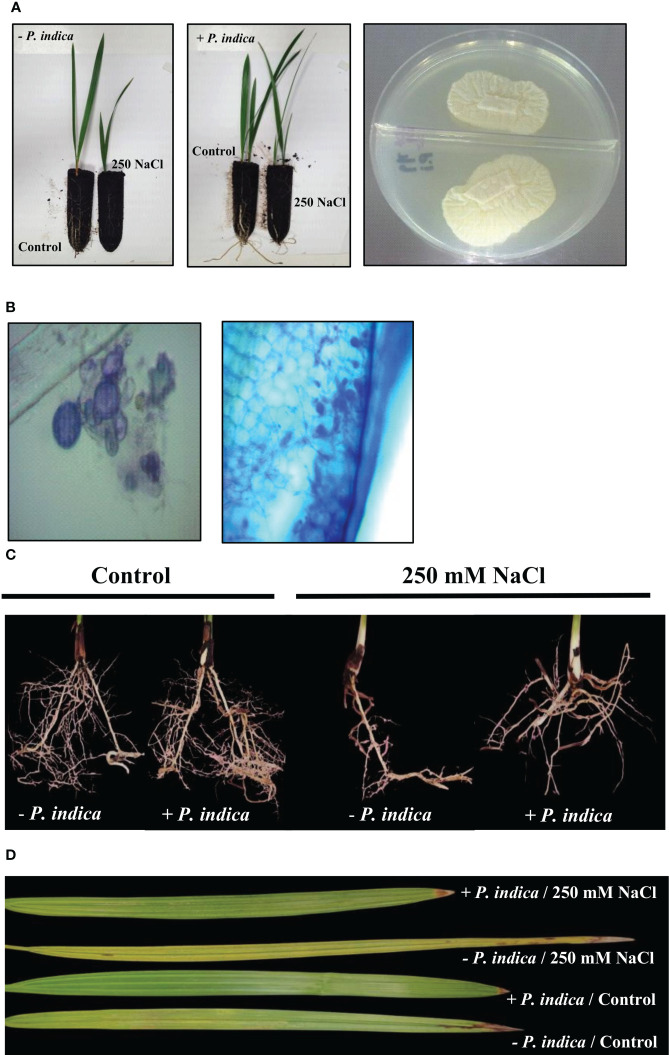
*P. indica* inoculation enhanced date palm seedlings root growth and biomass under control and salt stress conditions. **(A)** *P. indica* was axenically grown on PDA medium and used to inoculate date palm roots. **(B)** The colonized roots were stained and viewed under compound bright field microscope to confirm the inoculation. **(C)** Lateral root density of inoculated and non-inoculated date palm seedlings grown in pots under control and salinity stress conditions. **(D)** Detached leaves variation for inoculated and non-inoculated date palm seedlings under control and salt stress conditions.

After 20 days of growth in soil pots, physiological variations were observed in the salt stress *P. indica* inoculated and non-inoculated date palm plants (**Figure 1A**). The stained samples with *P. indica* displayed fungal mycelium colonization into the root surface of date palm seedlings, which penetrates into the root cortex 20 days after inoculation (**Figure 1B**).

**Figure 2:**
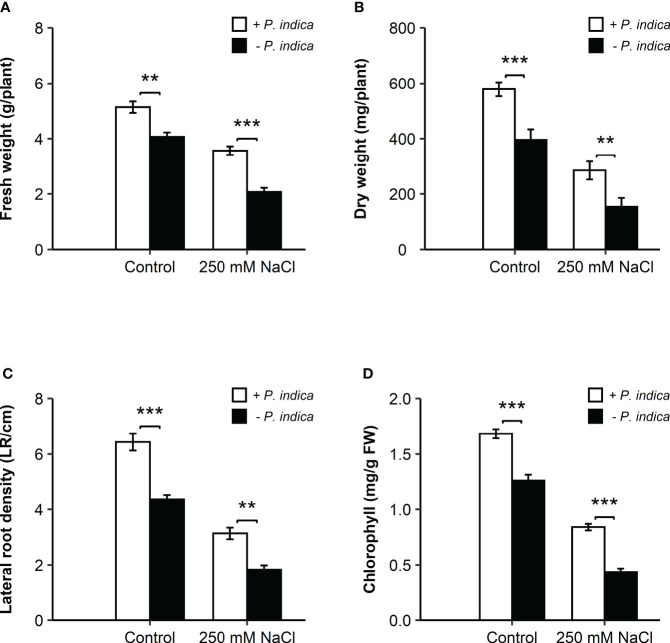
Effects of *P. indica* inoculation on the growth of date palm seedlings under normal (0 mM NaCl) and salt stress (250 mM NaCl) conditions. **(A)** Fresh weight (g/plant) of the inoculated and control plants. **(B)** Dry weight (mg/plant) of inoculated and control plants. **(C)** Lateral root density (LR/cm) of the inoculated and control plants. **(D)** Chlorophyll content (mg/g FW) of the inoculated and control plants. The values are the means ± SD (n = 3), analyzed by Student’s t- test, **p < 0.01 and ***p < 0.001.

The non-inoculated plants growth rate was reduced under exposure to salt stress. However, inoculation with *P. indica* displayed a significant increase in the growth of date palm seedlings grown in pots under control and salinity stress conditions for 20 days, as identified through fresh and dry weight and lateral root density estimation (**Figures 1C, D**, **2A–C**). Based on the Student’s t-test, a significant difference was observed in terms of the effects of *P. indica* on the fresh weight of date palm seedlings for the control (p < 0.01) and samples subjected to salinity (p < 0.001) (**Figure 2A**). *P. indica* inoculation increased fresh weight by 27.5% and 75% in control and salt stress conditions, respectively, as compared to uninoculated plants. The highest fresh weight was recorded for *P. indica*-inoculated date palm seedlings under control condition. In relation to the dry weight, a significant difference was found between the inoculated and non-inoculated plants under normal (p < 0.001) and saline conditions (p < 0.01) (**Figure 2B**). Plant inoculation with *P. indica* enhanced dry weight by 46.2% and 85.1% in the control and salt-stressed plants, respectively. In addition, *P. indica* colonization increased root branching of the control and stressed seedlings. A significant difference in the densities of lateral roots of colonized and non-colonized plants was observed under control (p < 0.001) and salt stress conditions (p < 0.01), with increased lateral root density in the inoculated plants (**Figure 2C**). It was stimulated by 48.8% and 72.2% in control and stressed plants, respectively, compared with the non-colonized plants. Moreover, there was a significant difference in the chlorophyll content of *P. indica*-colonized and non-colonized plants under both treatment conditions (p < 0.001) (**Figure 2D**). Net chlorophyll content was significantly higher in plants inoculated with *P. indica* than in non-inoculated plants, enhanced by 33.3% and 95.3% in non-stressed and stressed conditions, respectively. Therefore, it was found that inoculated plants were less affected by salinity stress (**Figures 2A–D**). The improvement in plant growth indicates that *P. indica* colonization enhances date palm’s salt stress tolerance.

### Inoculation of *P. indica* regulates Na^+^ and K^+^ homeostasis in date palm seedlings

To counter salt stress, plants reduce toxic Na^+^ ion accumulation in the roots and the photosynthetic tissue or restrict its entry into the cells by increasing the concentration of intracellular K^+^ ions. To validate this strategy, the roots and leaves Na^+^ and K^+^ ion concentration was measured from the young date palm seedlings inoculated with *P. indica* or not, under control or salt stress conditions. Na^+^/K^+^ ratio, which is a strong selection tool to screen for salinity tolerance, was exploited to differentiate between Na^+^ and K^+^ uptake.

**Figure 3:**
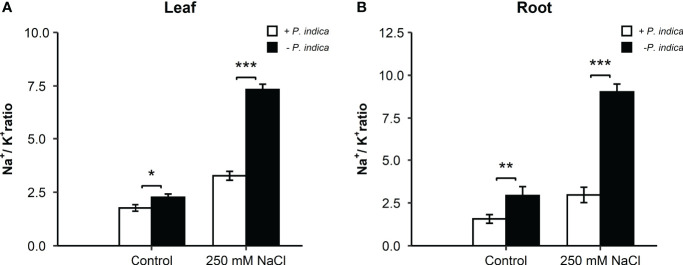
Effect of *P. indica* inoculation on the Na^+^/K^+^ ratio in date palm seedlings under control and salt stress conditions. **(A)** Leaf Na^+^/K^+^ ratio of *P. indica* colonized and non-colonized date palm seedlings. **(B)** Root Na^+^/K^+^ ratio of *P. indica* colonized and non-colonized date palm seedlings. The values are the means ± SD (n = 3), analyzed by Student’s t- test, *p < 0.05, **p < 0.01, and ***p < 0.001.

In date palm seedling leaves under control condition, a slight difference was observed (p < 0.05) in the Na^+^/K^+^ ratio of *P. indica*-colonized and non-colonized date palms (**Figure 3A**). Under salt stress conditions, a highly significant difference (p < 0.001) in the Na^+^/K^+^ ratio was observed in the leaves, with reduced levels in *P. indica*-colonized date palms (**Figure 3A**). Likewise, the Na^+^/K^+^ ratio declined significantly from the roots of *P. indica*-colonized date palm seedlings under control (P <0.01) and salinity stress (p < 0.001) (**Figure 3B**). Using this index, *P. indica*-colonized plants had a lower Na^+^ ion concentration compared to K^+^ ion in their leaves and roots. Under salinity stress conditions, colonized date palm seedlings had a two-fold and a one-fold reduction in Na^+^/K^+^ ratio in the leaves and roots, respectively (**Figures 3A, B**). Accordingly, reduced value of Na^+^/K^+^ ratio in plants growing under saline conditions in the presence of *P. indica* indicates its role in improving plants’ salinity stress tolerance (**Figures 3A, B**).

### *P. indica* colonization enhanced antioxidant enzyme activity in date palm seedlings challenged with salinity stress

**Figure 4:**
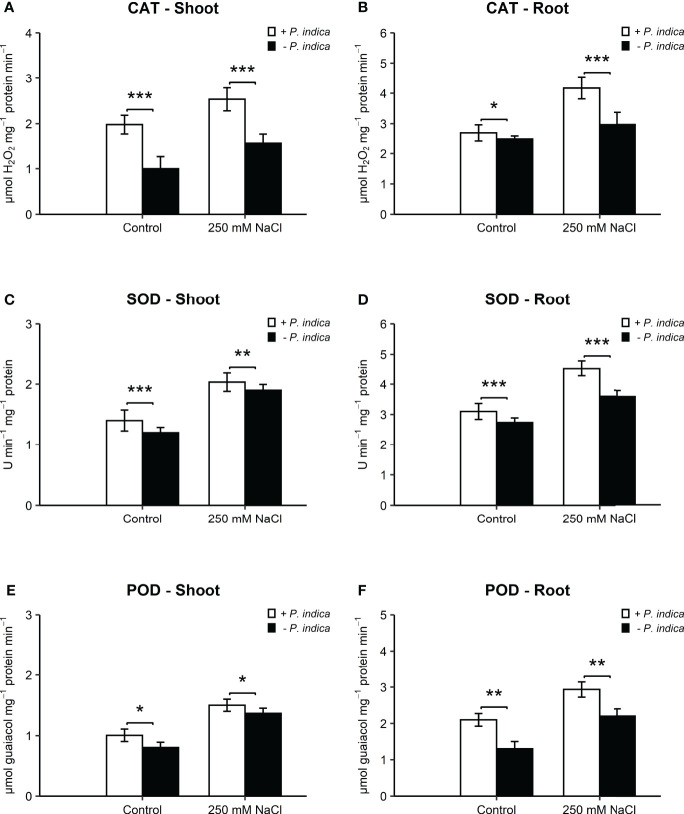
Antioxidant activity of date palm seedlings inoculated and non-inoculated with *P. indica* under control and salinity stress conditions. **(A)** Shoot content of CAT. **(B)** Root content of CAT. **(C)** Shoot content of SOD. **(D)** Root content of SOD. **(E)** Shoot content of POD. **(F)** Root content of POD. The values are the means ± SD (n = 3), analyzed by Student’s t- test, *p < 0.05, **p < 0.01, and ***p < 0.001.

The antioxidant enzyme activities of CAT, SOD, and POD were significantly different in the shoots and roots of colonized and non-colonized date palm seedlings under control and salt stress conditions (**Figures 4A–F**).

In the shoots, CAT activity showed an enhancement of 2- and 1.7-fold in *P. indica*-inoculated date palm seedlings under normal (p < 0.001) and salt stress (p < 0.001) (**Figure 4A**). In the roots, the increase in CAT activity was 1- and 1.6-fold in inoculated plants under control (p < 0.05) and salinity stress (p < 0.001) (**Figure 4B**). SOD activity recorded a significant increase of 1-fold in shoots of colonized seedlings as compared with non-colonized plants under normal (p-value < 0.05) and stress (p < 0.05) (**Figure 4C**). In the roots, SOD activity was enhanced by 1- and 1.7-fold in inoculated plants under control (p < 0.05) and salt stress (p < 0.01) (**Figure 4D**). POD activity was enhanced by 1.3- and 2-fold in inoculated plants under control (p < 0.05) and salt stress (p < 0.05) (**Figure 4E**). Root POD of *P. indica*-inoculated seedlings displayed an increase of 1.6- and 2.2-fold under normal (p < 0.01) and salinity stress (p < 0.01) (**Figure 4F**). The concentrations of the three antioxidant enzymes (CAT, SOD, and POD) in the shoots and roots of *P. indica*-inoculated date palm seedlings were the highest under salt stress.

### *P. indica* colonization increased Zn and P content in the roots of date palm seedlings

The root Zn and P concentrations were analyzed for determining the improvement in date palm nutrient concentration due to *P. indica*.

**Figure 5:**
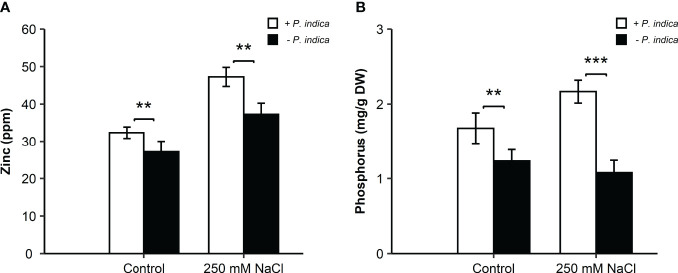
Zinc and phosphorus concentration in *P. indica* inoculated and non-inoculated date palm seedling roots under control and salt stress conditions. **(A)** Root zinc concentration (ppm) for *P. indica* colonized and non-colonized date palm seedlings. **(B)** Root phosphorus concentration (mg/g DW) of *P. indica* inoculated and non-inoculated date palm seedlings. The values are the means ± SD (n = 3), analyzed by Student’s t- test, **p < 0.01 and ***p < 0.001.

In the roots, under the control condition, there was a significant difference (p < 0.01) in the Zn concentration between the *P. indica*-colonized and non-colonized date palms, with a 1-fold higher concentration of Zn in *P. indica*-inoculated non-stressed date palm seedlings (**Figure 5A**). Likewise, under salt stress, a significant difference (p < 0.01) between the inoculated and non-inoculated plants was observed. The inoculated date palms seedlings had a 1.25-fold higher Zn concentration (**Figure 5A**). *P. indica* inoculation of roots increased the Zn content, compared to the respective controls. Moreover, in the roots, a significant difference under both the control (p < 0.01) and saline conditions (p < 0.001) was observed in terms of the P concentration between the inoculated and non-inoculated seedlings (**Figure 5B**). The P concentration showed an increase of 1- and 2-fold in the roots of *P. indica*-inoculated date palm seedlings under control and salt stress conditions, respectively (**Figure 5B**).

### *P. indica* regulates the transcript levels of some candidate genes (*PdHKT1;5*, *PdSOS1*, *PdRSA1*, *PdLEA2*)

**Figure 6:**
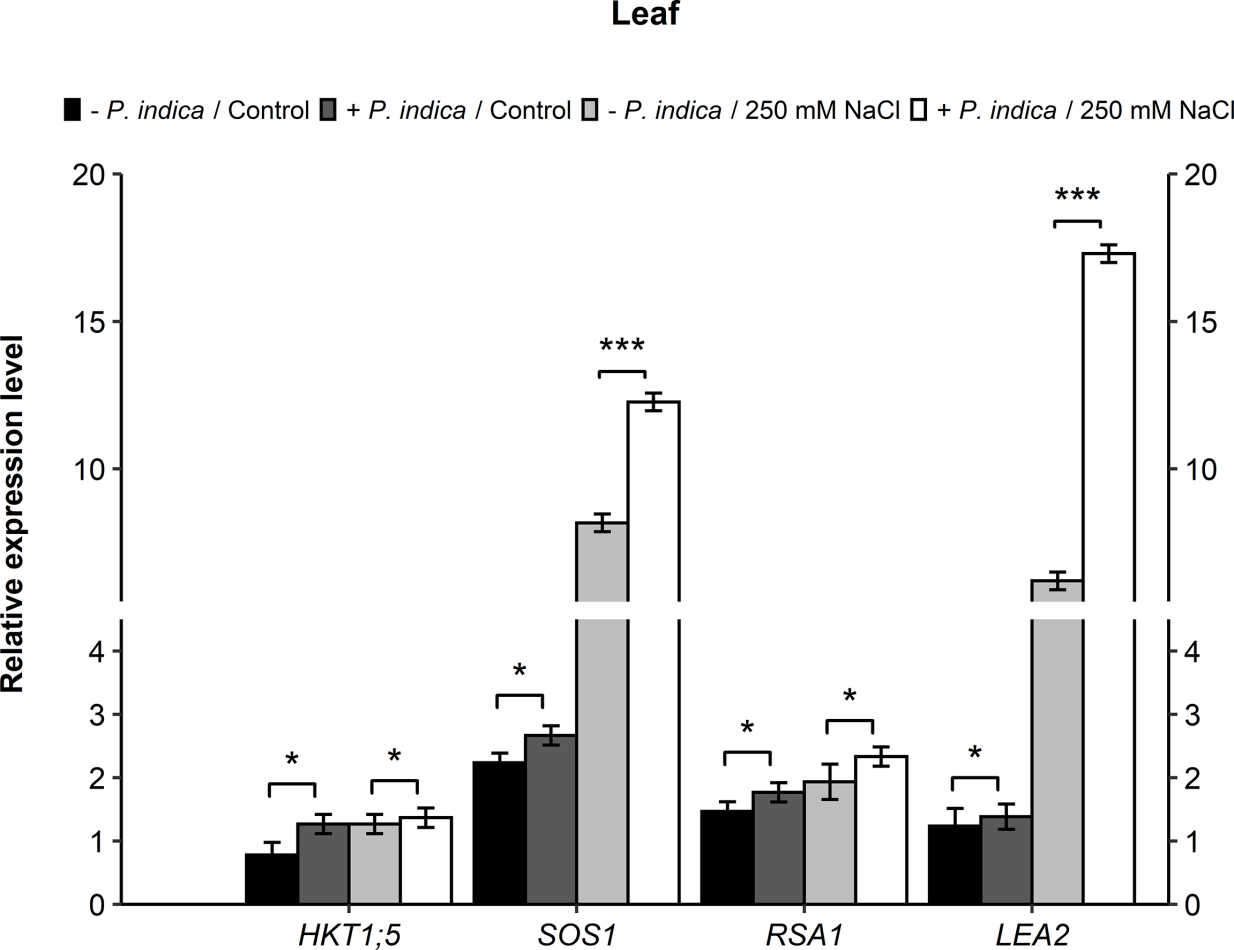
Relative expression of candidate genes (*HKT1;5, SOS1, RSA1,* and *LEA2*) in leaves of *P. indica* inoculated date palm seedlings grown under control and salt stress conditions. The values are the means ± SD (n = 3), analyzed by Student’s t- test, *p < 0.05 and ***p < 0.001.

To study the molecular aspects of salt stressed date palm seedlings inoculated with *P. indica*, the relative expression of various candidate genes encoding for stress-responsive proteins that play a role in Na^+^ and K^+^ homeostasis, were analyzed. Some of these included *HKT1;5* (high-affinity potassium transporter1), *SOS1* (Salt Overly Sensitive 1), *RSA1* (Short Root in Salt Medium 1), and *LEA2* (Late Embryogenesis Abundant) genes. Real-time PCR was performed on RNA samples from both inoculated and non-inoculated date palm seedlings under normal and salinity stress conditions. In relation to the expression level of the candidate genes in leaves, *HKT1;5* gene showed a slight difference (p < 0.05) in the expression of *P. indica*-colonized and non-colonized control and salt stressed date palm seedlings (**Figure 6**). *HKT1;5* gene expression was enhanced 1.6- and 1-fold for the control and stressed inoculated plants, respectively. For the *SOS1* gene, a slight difference in expression (p < 0.05) under control condition was observed for both the colonized and non-colonized date palm seedlings. Its expression under salt stress improved by 1-fold in *P. indica*-colonized plants. However, a significant difference was recorded in the *SOS1* level between the *P. indica*-inoculated and non-inoculated seedlings under salinity stress (p < 0.001) (**Figure 6**). An increase of 1.5-fold was observed in *SOS1* gene expression under salt stress in *P. indica*-inoculated seedlings. In the leaves, under normal and salt stress conditions, the expression level of *RSA1* gene showed a slight significant difference between the *P. indica*-colonized and non-colonized date palm seedlings (p < 0.05) (**Figure 6**)). An increase of 1.6-fold in *RSA1* expression was observed in the *P. indica*-inoculated date palm seedlings under salinity stress condition. Moreover, a slight significant difference (p < 0.05) was observed in *LEA2* gene expression under control condition between colonized and non-colonized date palm seedling leaves. However, a highly significant difference (p < 0.001) under salt stress condition was observed with 3-fold higher expression level in *P. indica*-inoculated seedlings (**Figure 6**).

**Figure 7:**
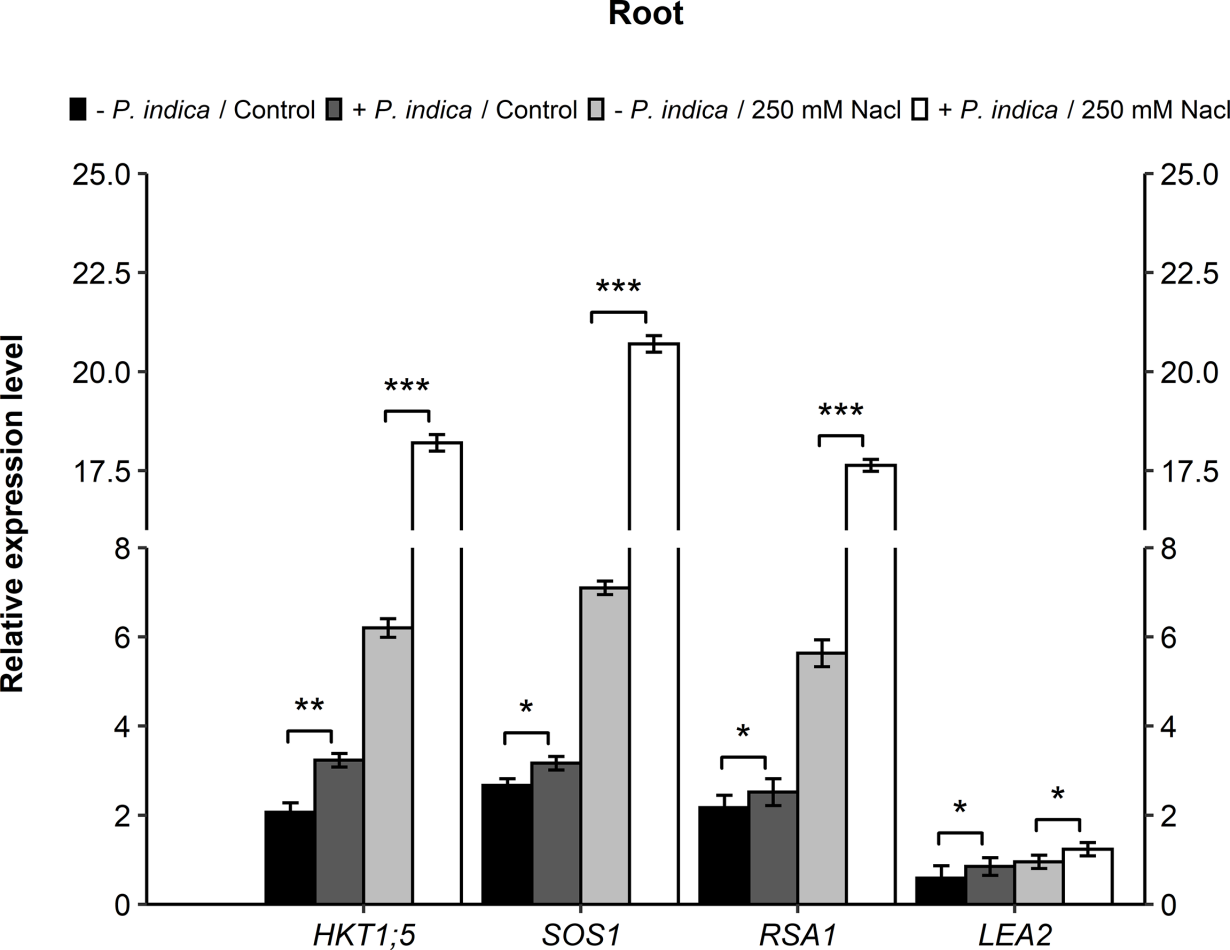
Gene expressions (*HKT1;5*, *SOS1*, *RSA1*, and *LEA2*) in roots of *P. indica* colonized and non-colonized date palm seedlings grown under control and salt stress conditions. The values are the means ± SD (n = 3), analyzed by Student’s t- test, *p < 0.05, **p < 0.01 and ***p < 0.001.

In contrast leaves, there was a significant difference of relative gene expression in the roots of stress and control *P. indica*-colonized and non-colonized date palm seedlings. In relation to the *HKT1;5* gene, a significant difference was recorded in the *P. indica*-colonized and non-colonized date palm seedlings under control (p < 0.01) and salinity stress (p < 0.001) conditions. There was a 1.6- and 3-fold increase in the *HKT1;5* gene expressions in *P. indica*-colonized control and stressed plants, respectively (**Figure 7**). Regarding the *SOS1* gene, a significant difference was observed in the expression level of *P. indica*-colonized and non-colonized date palm seedlings under control (p < 0.05) and salt stress (p < 0.001) conditions (**Figure 7**). In the roots, *SOS1* expression increased 1-fold under control and 3-fold under salinity-stressed for *P. indica*-inoculated seedlings (**Figure 7**). A significant difference was further observed in the expression level of *RSA1* gene in the *P. indica*-colonized and non-colonized date palm seedlings under normal (p < 0.05) and salinity stress (p < 0.001) conditions (**Figure 7**). In the roots, the *RSA1* expression level showed an increase of 1- and 3-fold under control and salt stress conditions, respectively. Moreover, *LEA2* genes displayed a slight significant difference under control (p < 0.05) and salinity stress (p < 0.05) conditions for the *P. indica*-colonized and non-colonized date palm seedling roots was recorded (**Figure 7**). An increase of 1.3- and 1.6-fold in the expression level of *LEA2* genes was observed in *P. indica*-inoculated seedlings under non-stressed and stressed conditions, respectively.

## Discussion

*P. indica* is known to enhance the growth of plant and its tolerance to biotic and abiotic stresses (Gill et al., 2016; Lin et al., 2019). Its role in increasing abiotic stress tolerance has been reported in various plant species (Baltruschat et al., 2008; Alikhani et al., 2013; Ghabooli et al., 2013), but not in date palm. Most studies have confirmed a mutualistic relationship between plants and *P. indica* after pre-cultivation that enabled the plants to tolerate stress. Prolonged exposure to salt stress causes ion toxicity due to sodium and chloride ion build up within the cytosol. This leads to a decline in potassium uptake, which affects the activity of various enzymes and impedes plant growth. Peskan-Berghöfer et al. (2015) demonstrated that increasing ABA triggered by osmotic stress promotes fungal colonization of the roots, without impairing plant fitness. This shows that ABA can strengthen plant interaction with *P. indica* as a consequence of its effect on plant innate immunity. However, the basis of the mechanism through which plant growth is improved by *P. indica* is not clear. Hence, in this study, *P. indica’s* ability to enhance date palm seedling growth is investigated through its impact on plant Na^+^ and K^+^ ion concentrations and expression of some genes and ion channels that maintain the Na^+^/K^+^ homeostasis and the nuclear-localized calcium-sensing and signaling pathway, which is also an essential mechanism of plant stress tolerance.

The date palm seedlings’ growth was impaired by the inclusion of salt in the growth media. However, the addition of *P. indica* countered these effects in the seedlings, resulting in increased biomass and lateral root density under control and salinity stress conditions. These results are in concurrence with previous studies of plant growth stimulation with *P. indica* inoculation under salinity (Ghorbani et al. 2018; Abdelaziz et al., 2019; Bertolazi et al., 2019; Atia et al., 2020). Likewise, under drought stress, it was found that *P. indica* improved the biomass and morphological characteristics of *Solanum melongena* (Swetha and Padmavathi, 2020). Furthermore, *P. indica* enhanced the chlorophyll content of inoculated date palm, as exhibited by their improved fresh and dry weight, both under control and stressed conditions. Similarly, enhanced chlorophyll content was recorded in *P. indica*-colonized *Arabidopsis thaliana* and rice plants (Jogawat et al., 2013; Abdelaziz et al., 2017). The colonization with *P. indica* improves the root architecture of plants that increases root surface area, which leads to improved plant biomass and chlorophyll content due to the efficient absorption of several ions such as the K^+^ and Ca^2+^ (Liu et al., 2019).

The leaves and roots of salt-stressed non-colonized date palm seedlings displayed a higher Na^+^/K^+^ ratio, possessing higher Na^+^ and lower K^+^ contents. On the other hand, *P. indica* colonization of salt-stressed date palm seedlings altered Na^+^/K^+^ homeostasis and resulted in a decline in the Na^+^/K^+^ ratio in leaves and roots. In accordance with the current results, it has also been reported that *P. indica*-colonized barley cultivated under 300 mM NaCl had enhanced K^+^ and lower Na^+^ ion content in the leaves, as compared with non-colonized plants (Deshmukh et al., 2006). Furthermore, decline in the Na^+^ content with the *P. indica* co-cultivation of Arabidopsis and barley were also reported under salt stress (Lanza et al., 2019; Sepehri et al., 2021). In relation to the present study, increased K^+^ ion content in the shoots of tomato plants inoculated with *P. indica* was also associated with its salinity tolerance (Yun et al., 2018). Cytosolic K^+^ homeostasis is considered an essential adaptation mechanism for salt stress in various plant species (Zhao et al., 2020). Thus, it can be suggested that the favorable Na^+^/K^+^ ratio modulation in colonized plant leaves and roots is associated with *P. indica’s* ability to preserve K^+^ retention under salinity.

Salt stress causes an increase in free radicals and ROS, which damages plant cells (Ma et al., 2022). The present findings show that *P. indica*-inoculated date palm seedlings had remarkably higher levels of CAT, SOD, and POD enzymes under salt stress conditions in comparison with non-inoculated seedling’s roots and shoots. These results suggest that *P. indica* inoculation enhances the triggering of ROS scavengers, which contributes to ameliorated salinity stress tolerance in date palm seedlings. In addition to ROS scavengers, it was further reported by Mohsenifard et al. (2017) that two miRNAs (miRNA396 and miRNA159) involved in the regulation of ABA, were significantly upregulated after inoculation of rice plants with *P. indica* under drought stress condition. It is known that miRNAs are key factors in regulating many biological processes by degradation or translation inhibition of stress-related genes. Similarly, under salt stress, *P. indica* can significantly increase ABA that stimulates the stomatal closure and prevents damages to the plants. Furthermore, previous studies have also shown that *P. indica* inoculation causes a rise in detoxifying and antioxidant enzymes level in the host plant species (Baltruschat et al., 2008; Matsuo et al., 2015). As observed in this study, *P. indica* also contributed to the reduced symptoms of stress in the date palm seedlings under salinity stress. Consistent with present study’s results, Lin et al. (2019) reported that CAT, SOD, and POD activities, which are crucial to the antioxidant activity of plant cells, were enhanced by the fungus. Moreover, under water stress condition, similar effects of *P. indica* were found in rice plants, with an increase of CAT enzymatic activity (Tsai et al., 2020).

The mineral analysis profile indicated that the symbiotic association between *P. indica* and date palm seedlings enhanced the P and Zn content under normal and salt stress conditions. The current study focused on the P and Zn uptake due to their essential role in maintaining plant growth and development under salinity. *P. indica* solubilizes organic P from the soil to make it readily available as Pi, that constitutes an indirect Phosphate source for the plants. In exchange, *P. indica* derives photosynthates (carbon), which is essential for its metabolic processes and survival, from the plants (Aslam et al., 2019). The increase in plant rhizosphere macro- and micronutrients due to *P. indica* association has been described in several plants (Gil et al., 2016; Su et al., 2017). Adequate amount of P in the soil enhances plant tolerance to excess salts by instigating a deeper rooting system and providing sufficient inorganic P supply for the absorption of carbon in leaves. It also plays a role in preserving optimal leaf relative water content under high salinity (Tariq et al., 2017). Comparably, Zn is also crucial for date palm’s growth under saline conditions due to its role in regulating antioxidative enzymes such as CAT and SOD. Thus, higher Zn concentration in the soil also helps plants to avoid ROS damage under salinity stress. The impact of *P. indica* colonization on P and Zn levels detected in the current study has also been expressed in lettuce plant species (Padash et al., 2016). PHOSPHATE1 (PHO1) is a high-affinity Pi transporter expressed predominantly in the roots, and the gene is up-regulated under low Pi conditions (Hamburger et al., 2002). Bakshi et al. (2015) reported that *P. indica* promotes PHO1 expression under Pi limitation.

This study found that *P. indica* inoculation increased seedling biomass and the number of lateral roots, altered Na^+^/K^+^ homeostasis, enhanced the activation of ROS scavenging enzymes and allowed for efficient mineral uptake in salt-stressed date palm seedlings. Furthermore, in a recent study, RNA-Seq experiment was performed for gene expression differences in Arabidopsis plants inoculated with *P. indica*. It was reported that in response to salt stress, the number of downregulated genes were higher in comparison to the upregulated genes. The *de novo* assembly of *P. indica* transcriptome generated a total of 15,410 unigenes, out of which, there were 661 genes differentially expressed against the salinity stress (Nivedita et al., 2021). To understand the molecular mechanisms through which *P. indica* enhances plant’s stress tolerance, a transcriptome analysis using RNAseq technology was performed on the roots of colonized and non-colonized date palm seedlings under salt stress and control conditions (data not shown). The up-regulated genes identified by RNAseq and those that were significantly enriched with transcripts involved in membrane polarization, ion transportation, and ROS signaling were investigated by real-time PCR. The results displayed that *P. indica* inoculation led to a three-fold increase in *HKT1;5*, *SOS1*, and *RSA1* transcript levels in the roots of inoculated plants as compared with non-inoculated plants under salt stress. The high *HKT1;5* and *SOS1* expression allows for the maintenance of a balanced Na+ and K+ ion homeostasis under salinity, which improves plant growth. Similar enhanced expression of *HKT1* and *SOS1* were found in *Arabidopsis thaliana* and *Lycopersicon esculentum* plants colonized with *P. indica* (Abdelaziz et al., 2017; Ghorbani et al., 2019). The HKT1;5 gene encodes a plasma membrane Na^+^ transporter expressed in the root cells surrounding xylem vessels (stele and epidermis), which is involved in Na^+^ retrieval from the xylem sap upon salt stress to reduce Na^+^ transfer to the leaves (Kiani et al., 2021). In rice, the Na^+^ transporter, OsHKT1;5, was expressed in vascular tissues (essentially in root xylem parenchyma), that retained Na^+^ ions in roots upon salt stress and enabled the shoot tissues to maintain K^+^ homeostasis. While the importance of SOS1 antiporter in plant salt tolerance is linked to Na^+^ homeostasis, that removes Na^+^ out of the cytosol into the apoplastic space and controls the root-to-shoot Na^+^ transport for the maintenance of Na^+^/K^+^ homeostasis (Shi et al., 2002; Olías et al., 2009). In addition, *RSA1* was also highly expressed in the colonized roots challenged with salt stress. *RSA1* and its partner, *RITF1*, govern the transcription level of various genes that play a role in ROS detoxification under salt stress. *RSA1* further regulates the *SOS1* gene that encodes for a plasma membrane Na^+^/H^+^ antiporter crucial for salinity tolerance (Guan et al., 2013).

*LEA2* genes are of particular importance in date palm, and they are speculated to retain water molecules and prevent crystallization of cellular components under water deficiency resulting from drought, heat, and high salinity stresses. These genes are opulent in the *P. dactylifera* genome assembly; there are 62 *LEA2* members in *P. dactylifera* as compared with 52 and 46 in rice and sorghum, respectively (Al-Mssallem et al., 2013). According to the genome assembly and the transcriptomic data generated from RNAseq carried out on date palm roots (data not shown), the current study analyzed the date palm’s *LEA2* genes expressions that regulate the plant’s responses to abiotic stress. The results indicated that *P. indica* inoculated led to a roughly three-fold higher *LEA2* transcript levels in leaves but not in inoculated plants roots contrast to non-inoculated plants under salt stress. In accordance with this result, Azizi et al. (2021) had reported that the interaction effect of drought and colonization of tomato seedlings with *P. indica* significantly enhanced the *TAS14* gene expression, one of the essential groups of *LEA2* genes.

## Conclusion

The salinity stress negatively impacts the agronomical traits of the date palm due to the accumulation of toxic Na^+^ ions in the plant cytosol. In fact, Na^+^ accumulation can damage membrane systems and cytosolic proteins. To eliminate these toxic Na^+^ ions and maintain ion homeostasis at the plasma membrane, efficient Na+ and/or K+ transport systems are required. The current study revealed that the colonization of date palm seedlings with the beneficial endophyte *P. indica* significantly reduced the detrimental effects of salt stress by exhibiting enhanced growth through ions homeostasis and nutrients uptake, antioxidant activity, and several upregulated stress responsive genes. Date palm root colonization by *P. indica* is a remarkable example of a beneficial microbial symbiosis and the underlying molecular mechanisms by which it establishes itself and exerts its beneficial effects are poorly understood. To increase our understanding of the mechanisms underlying the ability of this endophyte to promote growth and increase salt stress tolerance, we need to advance our understanding of the colonization mechanism and how the host responds transcriptionally to colonization. The profound symbiotic association between the *P. indica* and date palm seedling found in the present study suggests that this endophytic fungus can be exploited as an essential plant symbiont for improving date palm production for economic sustainability in marginal environments.

## Data availability statement

The original contributions presented in the study are included in the article/supplementary material. Further inquiries can be directed to the corresponding author.

## Author contributions

KM, MA, and MS conceived and wrote the original manuscript. SKM, MS, and MA performed the experiments. KM, MA, and MS analyzed the data. KM, MS, MA, SKM, FB, and HR reviewed and edited the draft. All the authors have read and approved to the submitted version of the manuscript.

## Funding

This research work was supported by funding from the United Arab Emirates University, the Research Office to K.M. under grant number 31R203.

## Conflict of interest

The authors declare that the research was conducted in the absence of any commercial or financial relationships that could be construed as a potential conflict of interest.

## Publisher’s note

All claims expressed in this article are solely those of the authors and do not necessarily represent those of their affiliated organizations, or those of the publisher, the editors and the reviewers. Any product that may be evaluated in this article, or claim that may be made by its manufacturer, is not guaranteed or endorsed by the publisher.
